# BiLSTM- and CNN-Based m6A Modification Prediction Model for circRNAs

**DOI:** 10.3390/molecules29112429

**Published:** 2024-05-21

**Authors:** Yuqian Yuan, Xiaozhu Tang, Hongyan Li, Xufeng Lang, Yihua Song, Ye Yang, Zuojian Zhou

**Affiliations:** 1 School of Artificial Intelligence and Information Technology, Nanjing University of Chinese Medicine, Nanjing 210023, China; 20211075@njucm.edu.cn (Y.Y.); hyli@njucm.edu.cn (H.L.); langxufeng@njucm.edu.cn (X.L.); yihua.song@njucm.edu.cn (Y.S.); 2 School of Medicine & Holistic Integrative Medicine, Nanjing University of Chinese Medicine, Nanjing 210023, China; tangxz1130@163.com

**Keywords:** CNN, BiLSTM, circRNAs, m6A

## Abstract

m6A methylation, a ubiquitous modification on circRNAs, exerts a profound influence on RNA function, intracellular behavior, and diverse biological processes, including disease development. While prediction algorithms exist for mRNA m6A modifications, a critical gap remains in the prediction of circRNA m6A modifications. Therefore, accurate identification and prediction of m6A sites are imperative for understanding RNA function and regulation. This study presents a novel hybrid model combining a convolutional neural network (CNN) and a bidirectional long short-term memory network (BiLSTM) for precise m6A methylation site prediction in circular RNAs (circRNAs) based on data from HEK293 cells. This model exploits the synergy between CNN’s ability to extract intricate sequence features and BiLSTM’s strength in capturing long-range dependencies. Furthermore, the integrated attention mechanism empowers the model to pinpoint critical biological information for studying circRNA m6A methylation. Our model, exhibiting over 78% prediction accuracy on independent datasets, offers not only a valuable tool for scientific research but also a strong foundation for future biomedical applications. This work not only furthers our understanding of gene expression regulation but also opens new avenues for the exploration of circRNA methylation in biological research.

## 1. Introduction

CircRNAs were first discovered in 1976 and identified as participating in regulating the progression of a variety of diseases [1]. The past 10 years have witnessed the advance of RNA sequencing technologies and computational methods for circular RNA annotation that unveiled widespread expression of circRNAs in various metazoan cell types and tissues [2,3]. Crucially, due to the special closed structures, circRNAs are stable, with half-lives ranging from 18.8 to 23.7 h (compared with 4.0–7.4 h for their cognate linear RNAs) and emerging evidence has shown that circRNAs are enriched in human tissues and body fluids including plasma, saliva, and urine, indicating that circRNAs are promising biomarkers in human diseases [1,2].

RNA modifications play a key role in modulating gene expression and functions. According to MODOMICS, more than 160 chemical modifications have been identified, opening up a new field known as “epitranscriptomics” [4,5]. CircRNAs suffer from a variety range of post-transcriptional modifications, including N1-methyladenosine (m1A), N6-methyladenosine (m6A), and 5-methylcytosine (m5C) [1]. Recent evidence suggests that post-transcriptional modifications of circRNAs play a critical role in various cellular processes. For example, as the most important RNA modification, m6A modification involves methylation modification of the nitrogen at the 6th position of the adenosine base, which performs a unique function in biological processes including circRNA splicing, translation, RNA-structure dynamics, subcellular localization, degradation, and cell reprogramming [6]. Notably, m6A-modified circRNAs demonstrate extensive influence in crucial areas like oncology, immune regulation, and neurological disorders. Their impact in these fields holds far-reaching implications. Chen et al. [7] made a seminal contribution by highlighting the critical role of m6A modifications in human endogenous circRNAs in modulating the innate immune response. They revealed that the modifications play a pivotal role in suppressing RIG-I activation, which is a key player in immunity. This finding opened new avenues for understanding the intricacies of immune regulation and the potential for therapeutic interventions in immune-related disorders. In addition, circRNAs could in turn regulate m6A modification and further affect the expression of target genes, which bring totally new insights into disease treatment [3]. 

Many scientists have concentrated on the prediction of m6A modification of mRNAs. Computational methods can be combined with experimental identification of mRNA modification sites, and significant strides have been made in both detection methodologies and the development of comprehensive databases. Techniques like m6A-seq and MeRIP-seq initially paved the way for m6A site identification, primarily at a transcriptome-wide level. Later, advancements such as miCLIP-seq refined this approach, enabling precise localization of m6A sites at the single-nucleotide scale. Presently, high-throughput sequencing technologies, including MeRIP-seq, miCLIP, m6A-REF-seq/MAZTER-seq, and DART-seq, stand at the forefront of m6A detection, marking a new era of RNA epigenetics research [8]. Efforts have particularly concentrated on enhancing the accuracy of tissue-specific predictions [9,10]. For instance, iRNA-m6A and m6A-binp, introduced by Dao et al. [11] and Wang et al. [12], respectively, utilize support vector machine-based approaches for predicting m6A sites across diverse tissues. Liu et al. [13] further advanced the field with the im6A-TS-CNN model, leveraging a single-layer convolutional neural network to achieve improved AUC values. Zhang et al. [14] developed a deep neural network (DNN)-based tool, DNN-m6A, which showed promising performance in identifying m6A sites across various tissues. Despite these advancements, certain challenges persist in the field of m6A methylation site prediction. 

An obvious shortcoming is the lack of focus on circular RNA; most prediction studies have concentrated on linear RNA, while circular RNA remains largely unexplored owing to insufficient experimental data that could serve as a knowledge base for analysis or predictive algorithms. However, there have been some attempts to address this gap, as seen in tools like CRIP [15], DeepciRGO [16], 3dRNA [17], cRNAsp12 [18], and other circRNA identification and prediction methodologies [19,20]. Additionally, many current algorithms exhibit suboptimal prediction accuracy in specific tissues. Another issue is the inherent limitations of existing techniques, such as a high rate of false positives, an inability to achieve single-nucleotide resolution, and challenges in accurately quantifying the modification stoichiometry [21,22]. In this work, we present a novel and robust predictive model for identifying m6A modifications in circular RNAs (circRNAs) based on the HEK293 cells dataset. This model leverages the complementary strengths of a bidirectional long short-term memory etwork (BiLSTM) and a convolutional neural network (CNN) to comprehensively analyze circRNA sequences. By integrating these powerful techniques, we not only address a critical gap in circRNA m6A prediction but also strive to significantly improve prediction accuracy compared with existing methods [23]. This innovative approach represents a substantial advancement in the field of RNA epigenetics, paving the way for more precise and insightful studies of m6A modifications within circRNAs.

## 2. Results

Since there are relatively few almost no methods for m6a methylation prediction for circRNAs, our method is based on the improvement and innovation of several existing methods for m6A methylation site prediction of linear RNAs in human gene tissues, combining the characteristics of circular RNAs and improving the model, which has yielded good results; therefore, only the comparative results of human gene tissues targeting circRNAs are reported in this paper.

### 2.1. Predicted Results for Human Genes

In this paper, a model for predicting m6A methylation of circular RNA (circRNA) was developed and tested on two different datasets, and the results are shown in Table 1. Dataset 1 was derived entirely from high-throughput sequencing experiments conducted in our laboratory, specifically designed to identify m6A methylation sites within circRNAs from human HEK293 cells. The dataset consists of a comprehensive collection of 1927 unique circRNA sequences. The results showed that the model demonstrated high accuracy and precision on its own independent dataset 1, with an accuracy of 78.18%, a precision of 76.27%, and an F1 score of 0.7895. The sensitivity of the model for positive samples (SN) was 81.82%, while the specificity for negative samples (SP) was 74.55%. The AUC of the model’s composite evaluation metric was 79.34%, and the Matthews correlation coefficient (MCC) was 0.5651. 

Since the prediction model in our work was developed from human genes, in order to maintain a continuous focus on human genes and to verify the validity of the model in different data sources, we have chosen to build a validation dataset 2. Dataset 2 is from the established TransCirc database (https://www.biosino.org/transcirc/, accessed on 20 December 2023), which includes 730 circRNA sequences that also feature the m6A methylation site in human genes. This allows the model to maintain generality and performance consistency over independent data reflecting similar biological conditions, thereby improving model reliability. Using dataset 2, the model similarly demonstrated good performance with an accuracy of 77.03%, a sensitivity/recall of 66.67%, and a precision of 78.57%. The F1 score is slightly lower than that of dataset 1, at 0.7213. The model has a higher specificity for negative samples using this dataset, at 85.37%, compared with an AUC of 82.19% and an MCC of 0.5333. These results indicate that the model constructed in this paper has a reliable performance and shows good stability and accuracy on different datasets.

### 2.2. Ten-Fold Cross-Validation of ROC Curves

**Figure 1:**
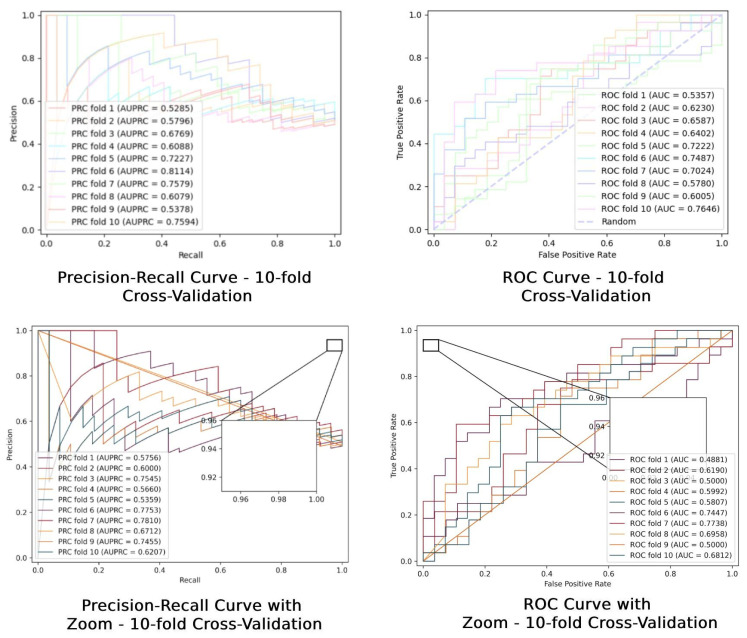
Ten-fold cross-validation ROC plots.

In this paper, model performance is evaluated by 10-fold cross-validation on an independent dataset. Overall, the model performance fluctuates across validation folds, which is reflected in differences in area under the curves (AUC and AUPRC) values. AUC (Area Under the Curve) refers to the area under the receiver operating characteristic (ROC) curve. It illustrates the diagnostic ability of a binary classifier system as its discrimination threshold is varied. ACC is the proportion of true results (both true positives and true negatives) in the population. It is a measure used to determine the overall correctness of a model and is calculated by dividing the number of correct predictions by the total number of cases examined. Both the precision-recall curve (PRC) and the receiver operating characteristic (ROC) curve show a consistent trend over multiple replicated trials, which indicates the stability and reliability of the model. The range of the AUC values shows the overall efficacy of the model in discriminating between the two categories (e.g., positives and negatives), whereas the curves in the PRC plots are more focused on the model’s precision in the prediction of positive samples. Overall, Figure 1 provides a comprehensive view of the diagnostic performance of the model and reveals the range of variation in specific performance metrics.

### 2.3. Modeling Summary

The model developed in this paper is a combination of a convolutional neural network (CNN) and a bidirectional long short-term memory network (BiLSTM). This hybrid model is designed to achieve high accuracy, exceeding 78%, in analyzing m6A methylation of circRNAs. The CNN part effectively captures local patterns and features in sequences, while the BiLSTM excels in processing sequence data and capturing long-distance dependencies. This combination allows the model to not only excel in identifying specific chemical markers but also to adapt to a wide range of different research environments and datasets. By integrating various performance metrics, such as accuracy, sensitivity, specificity, and AUC and MCC values, the present model not only performs well statistically but also has strong potential for practical applications. In addition, the application of 10-fold cross-validation further enhances the reliability of the model results and ensures its consistency under different experimental conditions.

### 2.4. Website Construction

The website employs best practices in modern web development, embracing the design principle of a decoupled front end and back end. The front end is developed using the Vue.js 3.0 [24] framework, which leverages reactivity and component-based architecture to deliver a highly interactive and user-friendly interface. For efficient data handling and separation of business logic, the back end is constructed using Python 3.9 [25], employing the Flask framework [26] to create a lightweight web service. Flask’s simplicity and flexibility enhance the speed and security of data transmission and processing. The seamless data interchange between the front end and back end is facilitated through a JSON-based RESTful API, enabling users to effortlessly upload circRNA sequences and receive predictions for m6A methylation sites.

**Figure 2:**
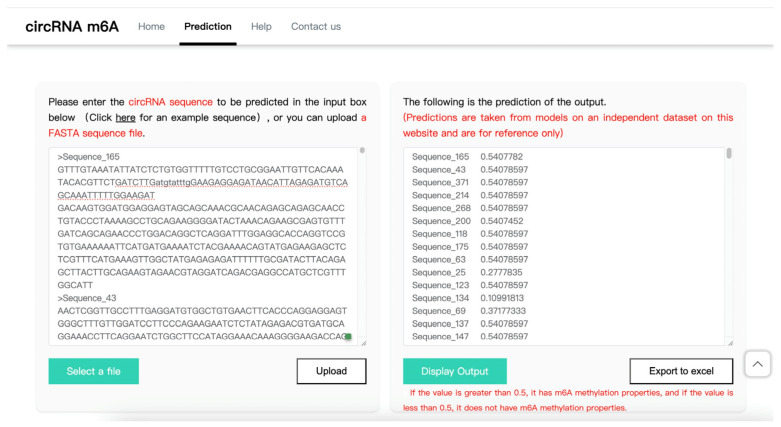
m6A methylation prediction interface for website circRNAs.

The site’s primary feature is an automated circRNA m6A methylation site prediction service, which holds significant value for research in bioinformatics. Precise m6A site prediction allows researchers to better understand the role of circRNAs in cellular regulation and to conduct in-depth analysis of disease mechanisms. Moreover, the site offers reference predictions based on an independent dataset, processed through specialized algorithmic models, the accuracy and reliability of which are acknowledged by the scientific community. The website prediction interface is shown in Figure 2. It is freely accessible at: http://ai.njucm.edu.cn:8081, accessed on 20 May 2024.

## 3. Discussion

m6A appears to play a critical role in various aspects of circRNA functions, including stability, subcellular localization, translation efficiency, phase separation, and alternative splicing. However, accurate identification of m1A and m6A sites within circular RNA remains a challenge. In this study, we present a novel sequence-based predictor for m6A sites in circRNAs. Building upon existing linear mRNA m6A prediction tools, our adapted model effectively identifies potential m6A modification sites. This predictor demonstrates promising performance, achieving an auROC of 0.78 on independent tests.

Numerous RNA modification site predictors have emerged, but their effectiveness varies significantly. One major limitation stems from training models on data lacking single-nucleotide resolution. This approach often leads to subpar performance when applied to datasets with higher resolution. Examples include RAMPred [27] and iRNA-3typeA [28], both developed using non-single nucleotide data and exhibiting limited success on independent high-resolution datasets. This highlights the need for robust predictors built upon data with single-nucleotide granularity. While some recent advancements, like SRAMP [29], Gene2Vec [30], and WHISTLE [31], addressed this issue by utilizing high-resolution data, their applicability remains restricted. These models are primarily designed for m6A locus prediction in specific species like Homo sapiens and Mus musculus.

In addition to dataset quality, dataset size is another important aspect to consider when training robust predictors. Some early forecasters could only use small datasets to train their models, which would result in poor predictive performance when tested on large datasets. For example, M6ATH [32] was built on the *A. thaliana* dataset, which contained only 394 m6A sites. The dataset was too small to cover the pattern of information sequences around the m6A locus. To ensure forecasters’ models remain current, retraining is recommended whenever a new dataset becomes available. Nearly all computational methods for predicting RNA modification sites rely on machine learning algorithms. To further enhance prediction accuracy, we propose two key strategies. Leverage sequence similarity: Methods successfully used for protein post-translational modification site prediction could be adapted for RNA analysis. Tools like GPS (Group-based Prediction System) demonstrate potential for extension into the realm of RNA modification site prediction [33]. Adopt integrated learning: Combining multiple approaches can offer superior results. For example, ZincExplorer, a zinc binding site predictor, demonstrates the power of integrating the outputs of three distinct predictors [34]. Notably, this strategy incorporates a sequence similarity-based approach for enhanced performance.

This work presents a novel circRNAs m6A methylation prediction model leveraging CNN and BiLSTM, offering significant advancements in the field of bioinformatics. This model addresses the challenge of efficient and accurate circRNA m6A site prediction by ingeniously combining advanced deep learning techniques. Specifically, the model extracts local sequence features through a CNN layer, captures long-range dependencies within the data using a BiLSTM layer, and employs an attention mechanism to prioritize critical information, significantly enhancing prediction accuracy and reliability. This approach holds not only fundamental importance for understanding m6A methylation’s role in circRNA function but also potential for future disease diagnosis and treatment strategies. Additionally, the model’s robust performance across diverse tissues and conditions establishes it as a powerful tool for m6A methylation studies. In conclusion, this study not only delivers a precise and efficient tool for m6A methylation site prediction but also opens new avenues for deep learning applications in bioinformatics, paving the way for its significant role in future genomics research.

## 4. Materials and Methods

### 4.1. Materials

**Figure 3:**
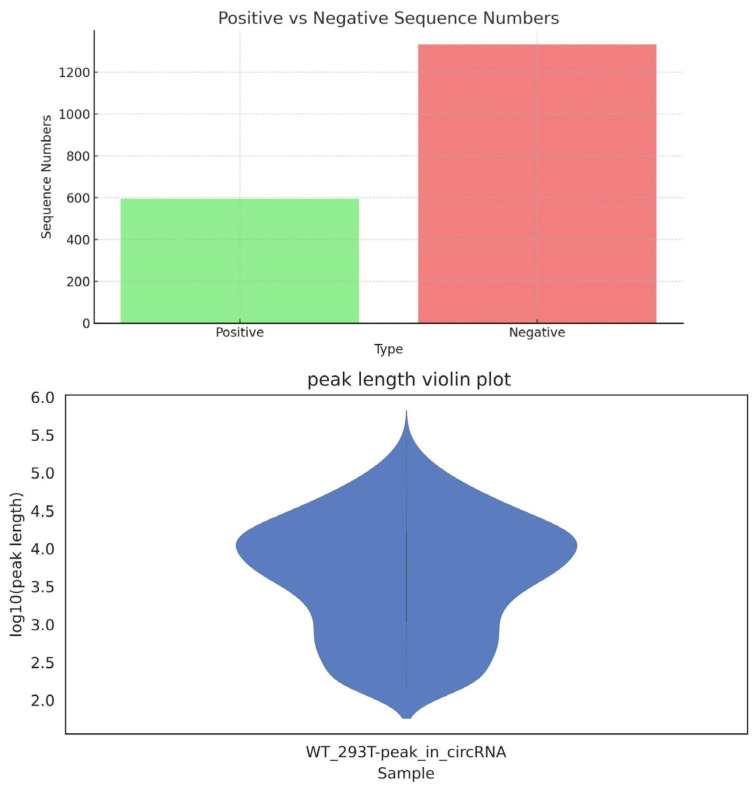
Distribution of m6A methylation in circRNAs from HEK293 human cells.

The dataset employed in our study was meticulously developed using high-throughput sequencing experiments carried out in our laboratory that were specifically designed to identify m6A methylation sites within circRNAs derived from human HEK293 cells. The resulting dataset comprises a comprehensive collection of 1927 unique circRNA sequences from human tissues, including 594 positive samples with m6A methylation and 1333 negative samples without methylation. Detailed statistical data for these samples are presented in Table 2, and their distribution is illustrated in the bar chart shown in Figure 3.

We divided the data obtained by high-throughput sequencing in our laboratory into two parts: a training dataset for model development and an independent test dataset for evaluating the model’s performance. Both subsets included positive samples (m6A sites) and negative samples (non-m6A sites), each standardized to the same sequence length. Given the substantial variation in sequence lengths within the original data—for example, the longest sequence in the positive samples was 83,698 nucleotides (nt), and the shortest was 159 nt, with an average length of 2437.49 nt and a median of 708.5 nt; in contrast, the negative samples ranged from 99 nt to 72,762 nt, with an average length of 1569.68 nt and a median of 504 nt—we strategically selected portions of the sequence set for the training dataset and the remainder for the test dataset to fine-tune the model parameters.

**Figure 4:**
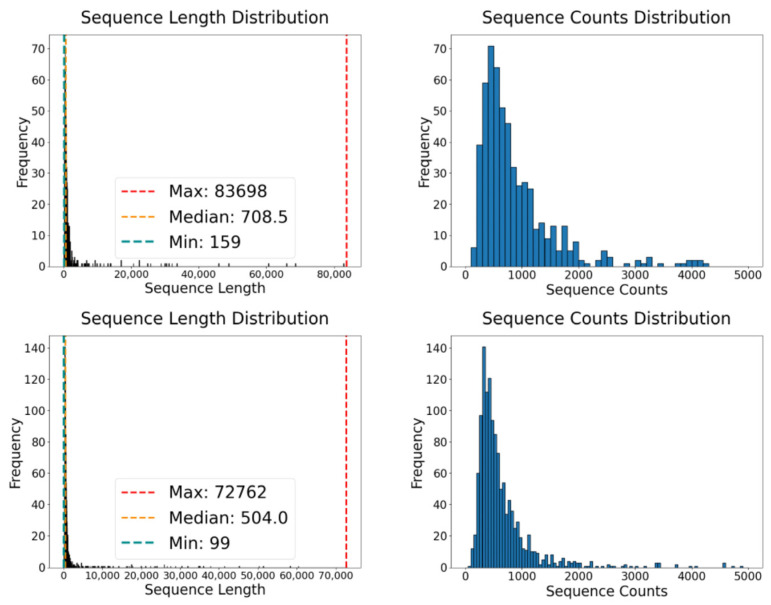
Statistical charts of data sequence lengths.

For the training and testing processes, we ensured that each sequence, whether positive or negative, was trimmed to 1000 nt, with adenine (A) positioned centrally. The training and test datasets were divided in a 7:3 ratio to balance the depth of learning with the integrity of performance validation. Specifically, 70% of the data were designated for training the model to ensure comprehensive learning across various scenarios present in the dataset. The remaining 30% were used as the independent testing set, enabling us to evaluate the model’s effectiveness and generalizability on new, unseen data. This ratio was strategically chosen to balance in-depth model training with rigorous performance validation. Detailed statistics on the sequence lengths in these datasets are provided in Figure 4.

**Figure 5:**
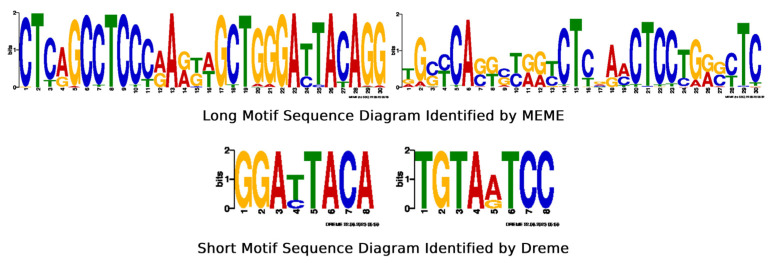
Motifs in peak sequences.

In biology, a motif represents a pattern in the distribution of nucleic acid sequences that holds biological significance. Within the realm of m6A research, a motif describes the specific arrangement of nucleic acids at sites undergoing m6A modification, which can be recognized and bound by RNA methylation-associated proteins to fulfill distinct biological functions. MEME-ChIP [35] was also chosen, as the data used in this paper came from high-throughput sequencing experiments performed in our laboratory, and MEME-ChIP is a versatile tool for identifying patterns in large datasets generated by high-throughput sequencing and is particularly suitable for analyzing epigenetic modifications such as m6A methylation in circRNAs [36,37]. In the m6A methylation prediction model for circRNAs, it is not difficult to find that MEME-ChIP helps to identify potential m6A methylation sites that may be critical for circRNA processing, localization, stability, or interaction with proteins. By integrating MEME-ChIP in the analysis of circRNA m6A sites, researchers can not only identify potentially novel motifs but also cross-reference these findings with known data to assess their relevance and impact on circRNA functionality. This dual approach of discovery and validation ensures a comprehensive understanding of the epigenetic regulation of circRNAs. Therefore, we employ MEME-ChIP to identify motifs within peak sequences, as illustrated in Figure 5.

### 4.2. Method

#### 4.2.1. Construction of Predictive Models

Considering the possible combinations of each of the four nucleotide bases in the circRNA sequences (i.e., adenine (*A*), cytosine (*C*), guanine (*G*), and uracil (*U*)), to represent each of the bases in the circRNA sequences as a fixed-length vector, capture the spatial information and relative position of the bases, and improve the prediction accuracy of the m6a locus in the different RNA sequences, we used the following single thermocode representation [38]:

$$A=(1,0,0,0{)}^{T}$$

$$U=(0,1,0,0{)}^{T}$$

$$C=(0,0,1,0{)}^{T}$$

$$G=(0,0,0,1{)}^{T}$$

The model used in this paper incorporates a convolutional neural network (CNN) and a bidirectional long short-term memory network (BiLSTM) with an attention mechanism to efficiently process and analyze sequence data. A convolutional neural network (CNN) is a deep learning algorithm that is widely used in image and video processing [39]. It is efficient in extracting local features while processing sequence data. A CNN works by sliding window convolution kernel moving along the data and applying filters, which help capture the local features of the data. A bidirectional long short-term memory network (BiLSTM) is a special type of recurrent neural network (RNN) that outperforms traditional RNNs in that it avoids the long-term dependency problem. BiLSTM enables the network to have contextual information in the forward and backward directions by running two independent LSTMs in both directions of the data, which, in the case of processing sequential data (e.g., text and speech recognition), is especially important [40]. Therefore, in this paper, we apply these two learning algorithms to process sequence data and extract useful features for m6a methylation prediction.

**Figure 6:**
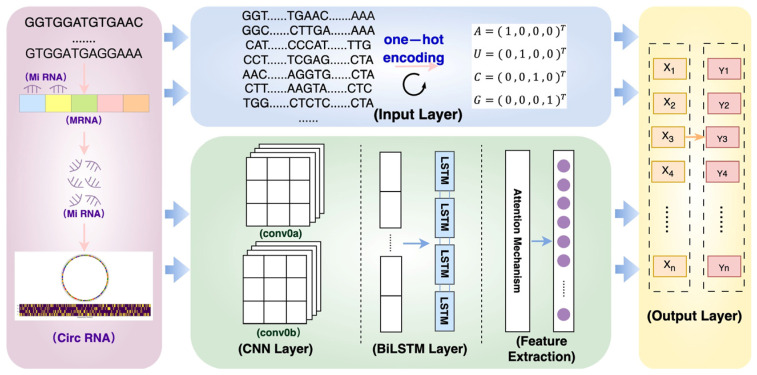
Framework diagram of circRNA prediction model based on Convolutional Neural Network (CNN) and Bidirectional Long Short Time Memory (BiLSTM).

Specifically, the model in this paper first extracts the local features of the input data through two CNN layers with different configurations, where the first convolutional layer employs 64 1 × 8 convolutional kernels with a step size of 3, while the second one uses 32 1 × 3 convolutional kernels with a step size of 2, with the application of batch normalization and ReLU activation functions after each convolution. The output of the CNN layer is then sent to the BiLSTM layer, which captures the long-term dependencies in the time-series data by applying LSTM units in each of the positive and negative directions of the sequence. The output of the BiLSTM layer is further weighted by the attentional mechanism, allowing the model to focus on the most critical information in the sequence. Ultimately, the overfitting is reduced, and the generalization ability of the model is improved through the fully connected layer and dropout technique, and the output layer uses a softmax function to generate predictions. This hybrid model combining CNN, BiLSTM, and an attention mechanism not only exploits the advantages of CNN in feature extraction but also combines the ability of BiLSTM to capture long-term dependencies, and at the same time enhances the identification of key information through the attention mechanism, demonstrating the cutting-edge technologies and methods of deep learning in the field of sequence data processing [41,42]. The design of this model reflects the latest advances in deep learning, BiLSTM, and attention mechanisms and is a major innovation in the field of circRNA sequence data analysis. The model framework of this paper is shown in Figure 6.

#### 4.2.2. Detailed Algorithm Flow

(a) The circRNA sequence data are read, and two feature representations are used: a single thermal encoding, in which each nucleotide (A, U, G, C) is encoded as a vector of length 4, and a conversion of the sequences into a dual encoding form that adds additional information for each nucleotide.

(b) Feature extraction of the input RNA sequence using two convolutional layers. The first convolutional layer uses 64 1 × 8 convolutional kernels with a step size of 3. This corresponds to learning patterns of length 8 in the sequence. The output of each convolutional kernel is batch normalized and activated by the ReLU activation function. The second convolutional layer uses 32 1 × 3 convolutional kernels with a step size of 2 to further extract sequence features.

(c) The BiLSTM layer is entered to capture the long-term dependencies in the sequence data. In BiLSTM, the hidden state $h_{t}$ at each time step is jointly determined by the previous state $h_{t}$ and the current input $x_{t}$, as in Equation (1):

(1) $$h_{t}=BiLSTM(h_{t-1},x_{t})$$

(d) The BiLSTM layer is followed by an attention mechanism that identifies the most important parts of the sequence to improve the predictive power of the model. The attention weights are calculated by comparing the output of the BiLSTM layer with the last hidden state, as in Equation (2):

(2) $$Attention\;Weight=softmax(W\cdot h_{t})$$

where W is the learnable weight matrix. The output of the BiLSTM layer is processed through a fully connected layer and a dropout layer to reduce the risk of overfitting and finally classified by softmax function.

(e) For model prediction probability calculation, in this paper, the predicted output of the model on the input data is represented by f(x). Specifically, f(x) is the output obtained by the model in the forward propagation process, which contains the original score or probability of each sample, as in Equation (3):

(3) $$f(x)=\frac{1}{1+e^{-x}}$$

In Equation (3), x is the output value of the previous flattened layer processing, and f(x) is in the range of [0, 1], which is equivalent to the probability value. When f(x) > 0.5, the prediction is a positive sample (with m6A methylation properties); when f(x) < 0.5, the prediction is a negative sample (without m6A methylation properties).

#### 4.2.3. Modeling and Parameterization

In this study, we are concerned with predicting m6A methylation modification sites, which is a binary classification task. In this paper, we construct a hybrid convolutional neural network (CNN) and bidirectional long short-term memory network (BiLSTM) model; i.e., the CNN_BiLSTM model. This structure utilizes the CNN to capture local patterns in sequences, while the BiLSTM is used to deal with long-term dependencies in sequences. To train this model, this paper uses cross-entropy loss, which is a commonly used loss function in classification problems [43], as in Equation (4):

(4) $$Loss=-\frac{1}{N}\sum_{i=1}^{N}\left[y_{i}\cdot log(p(y_{i}))+(1-y_{i})\cdot log(1-p(y_{i}))\right]$$

where $y_{i}$ is the true label, $p(y_{i})$ is the probability predicted by the model, and N is the number of samples.

The model is trained using the Adam optimizer [44], which combines the advantages of the AdaGrad [45] and RMSProp [46] optimizers to automatically adjust the learning rate. In addition, the StepLR scheduler is used to reduce the learning rate by a certain percentage after every 10 training cycles to help the model converge more stably. In order to prevent overfitting and improve training efficiency, an early termination strategy is implemented by setting the maximum patience period to 5, such that if the performance of the validation set does not improve within five consecutive training cycles, the training will be terminated early. In order to evaluate the model performance, this paper adopts a 10-fold cross-validation method, where the data set is divided into 10 parts, and one of them is used as the test set and the rest as the training set in turn, thus ensuring that each data point can be used for testing and improving the reliability of the evaluation. The modelling approach established in this paper provides a comprehensive understanding of the performance of the model on the task of m6A methylation site prediction and ensures that the model has good generalization ability and high accuracy.

#### 4.2.4. Evaluation Indicators

To evaluate the model, the main evaluation metrics used in this paper include accuracy, recall, precision, SN, SP, F1 score, and the Matthews correlation coefficient (MCC), as shown in Equations (5)–(11) below. These metrics provide a comprehensive view to evaluate the performance of the model in different aspects. In particular, accuracy is the proportion of correct predictions, recall measures are the model’s ability to identify positive examples, sensitivity (SN), also known as the true positive rate, measures the proportion of actual positives that are correctly identified by the model, specificity (SP), also known as the true negative rate, measures the proportion of actual negatives that are correctly identified as such [47], precision reflects the veracity of the samples predicted to be positive examples, the F1 score is a balanced measure of precision and recall, and the MCC is a performance metric that takes into account all the elements of the confusion matrix in a comprehensive manner [48]:

(5) $$Recall=\frac{TP}{TP+FN}$$

(6) $$Precision=\frac{TP}{TP+FP}$$

(7) $$SN=\frac{TP}{TP+FN}$$

(8) $$SP=\frac{TN}{TN+FP}$$

(9) $$F1=2\times \frac{Precision\times Recall}{Precision+Recall}$$

(10) $$Accuracy=\frac{TP+TN}{TP+TN+FP+FN}$$

(11) $$MCC=\frac{\left(TP\times TN\right)-\left(FP\times FN\right)}{\sqrt{\left(TP+FP\right)\times \left(TP+FN\right)\times \left(TN+FP\right)\times \left(TN+FN\right)}}$$

In addition, model performance is also assessed through receiver operating characteristic curves (ROCs) and precision-recall curves (PRC). The ROC curve evaluates the model by depicting the relationship between the rate of true instances and the rate of false positive instances, whereas the AUC (area under the curve) provides a way to quantify the overall performance of the model. The PRC is particularly important in unbalanced datasets because it focuses on the predictive performance of positive cases [49]. These evaluation metrics take into account all aspects of the model to ensure high accuracy and reliability in real-world applications.

## 5. Conclusions

This study presents a novel predictor for m6A methylation sites in circular RNA, employing a combination of CNN, BiLSTM, and an attention mechanism for enhanced accuracy. By addressing previous limitations of low-resolution data and small datasets, it achieves an auROC of 0.78, marking a significant advance in circRNA methylation site prediction. This tool’s high performance and innovative approach exemplify the potential of deep learning in bioinformatics, paving the way for future genomic research and applications in disease diagnosis and treatment.

## Figures and Tables

**Table 1:** Evaluation metrics on human independent test datasets.

Datasets	Acc	SN	SP	F1 Score	AUC	MCC
Independent dataset	78.18%	81.81%	74.55%	0.79	79.34%	0.57
TransCirc dataset	77.03%	66.67%	85.37%	0.72	82.19%	0.53

**Table 2:** Dataset of m6A methylation sites.

Term	All	Positive	Negative	≥500 nt	≥100 nt	N50	Max_ Length	Min_ Length	Average_ Length
circRNAs	1927	594	1333	1093	426	16,318	83,693	99	1837.18

## Data Availability

The data presented in this manuscript are available from the corresponding author upon request.
